# Analysis of Repeated CT Scan Need in Blunt Head Trauma

**DOI:** 10.1155/2013/916253

**Published:** 2013-12-03

**Authors:** Serkan Emre Eroglu, Ozge Onur, Sefer Ozkaya, Arzu Denızbasi, Hasan Demır, Cıgdem Ozpolat

**Affiliations:** ^1^Department of Emergency Medicine, Marmara University Pendik Research and Training Hospital, Üst Kaynarca, Pendik, 34890 Istanbul, Turkey; ^2^Department of Emergency Medicine, Fatih Sultan Mehmet Research and Training Hospital, 34752 Istanbul, Turkey

## Abstract

*Background*. Computed tomography (CT) is a vital tool in the workup of patients with closed head trauma. The aim of this study was to investigate the necessity of serial CT scans in patients with blunt head trauma. *Methods*. This is a retrospective study analyzing trauma patients between January and June 2012. Data were analysed by using frequencies, Kolmogorov-Smirnov (K-S), and Chi-square tests. *Results*. Of the total 351 control Head CTs, it was seen there were no different in 346 (98.6%). In CTs of another 3 patients (0.9%), there were increasing or new, in the other 2 (0.6%) there was a decrease in the pathology present. Of 24 (6.8%) patients who had a hemorrhage in the first CT, there was an increase in the hemorrhage in one of them, a decrease of the pathology in 2 of them. Of 27 (7.7%) patients who had fracture in first CT, 2 had a new intracranial hemorrhage. The relation of the results between the first and second CTs were statistically significant (*P* < 0.001, *χ* ^2^ test). *Conclusion*. Repeated CT scans after 6 hours in EDs observation rooms are not necessary if first CT is normal in most situations. Special attention may be needed in patients with an underlying chronic disease.

## 1. Introduction 

Blunt head trauma is a common pathology seen in emergency departments (EDs). Initial evaluation includes a careful neurological examination and computed tomography (CT) scans of the brain [1]. CT scans is the represent the initial study of choice in current practice to determine the type, extent and severity of traumatic brain injury as well as to determine the management protocol [2]. The role of the initial brain CT scan and of unscheduled repeat brain CTs when a neurological deterioration occurs is well established [3]. However, there are no guidelines on the necessity for or the value of a repeat CT scan. There are reports emphasizing the importance of serial CT scans in patients with head trauma, while others feel it to be unnecessary in most patients. Nevertheless, patients who present with head trauma often receive repeat CT scans to rule out the progression of their head injury.

The aim of this study was to determine whether serial CT scans are necessary to identify the incidence of delayed positive findings in patients who present to the ED with blunt head trauma.

## 2. Methods

This is a retrospective study analyzing trauma patients between January and June 2012 in Marmara University Pendik Research and Training Hospital. The study was approved by the Institutional Review Board.

In our emergency department, trauma patients of all ages are first seen by emergency physicians and residents in emergency medicine. Our clinic has a head trauma protocol. We apply the Canadian CT Head Rules for deciding whether to take a CT scan for all head trauma patients [4, 5]. If there is no need for imaging according to these rules, we send the patients home with cautions. If there is need for neuroimaging, then we send patients for a head CT scan and refer the patient to a neurosurgeon. The neurosurgeons decide whether to send the patient home, observe them in the emergency observation room, have a control cranial CT after 6 hours if there is no deterioration of the patient earlier, or admit them to the neurosurgery ward or intensive care unit.

There are lots of decisions for control CTs and all of the patients waiting 6 hours for control CT scans are awaiting in ED observation room. This study consists of retrospective analyzing dataset of these patients.

### 2.1. Inclusion Criteria

All patients with blunt head trauma who were admitted to the neurosurgery department and subjected to two or more CT scans of the brain were included in the study. The decision to repeat a CT scan was taken by the neurosurgeon.

### 2.2. Exclusion Criteria

The following patients were excluded from the study:

- patients who were not referred to a neurosurgeon,
- patients who died before the CT scan,
- patients who had a penetrating injury,
- patients with associated life threatening injuries to other systems or polytrauma,
- patients who were taken up to surgery based upon the findings of the first CT scan,
- patients who were discharged or who expired after the first CT scan,
- patients who had a repeat head CT due to nontrauma-related incidental findings (brain tumor, cysts).

The first CT scan of the brain was referred to as admission CT (CTa). CTa was done as soon as possible after the trauma.

There was no standard protocol for repeating the CT scan of the brain. It was ordered by the neurosurgeon after personal assessment of the patient.

The initial CT scans of head were performed on a General Electric high speed helical scanner without intravenous contrast. The radiologist's initial reading was used to determine whether the scan was considered positive. Images were reread by in-house neuroradiologist the next day to verify the initial interpretation. The repeat CT scan was considered positive if it showed any intracranial abnormality that was not previously demonstrated on the initial imaging study.

Details like age, sex, time, use of anticoagulant/antiplatelet, the findings on each CT, the type, site and number of intracranial lesions were recorded.

All data were collected by an emergency physician or by a supervised resident in the emergency medicine training program.

**Figure 1:**
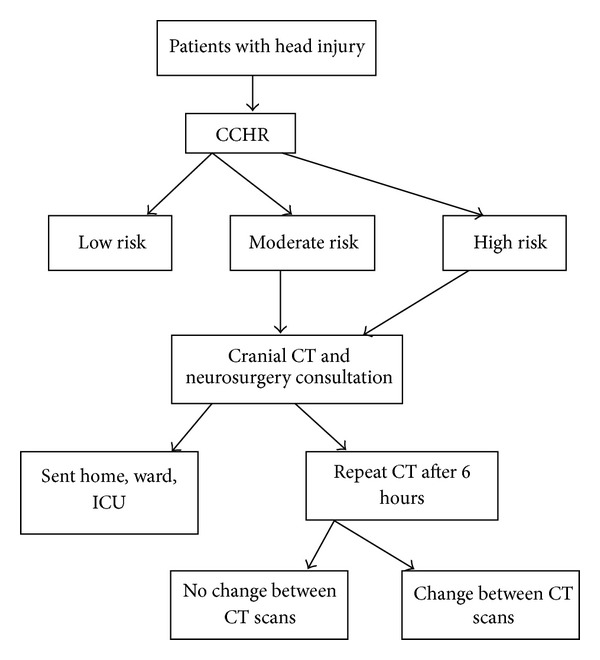
Study flow diagram (CCHR: Canadian CT Head Rule, CT: cranial tomography, and ICU: intensive care unit).

A study flow diagram is shown in Figure 1.

All statistical analyses were performed using SPSS v16.00 statistical analysis software. The average values are presented with 95% confidence interval (CI) in this study. The concordance of the relative variables to the normal distribution was evaluated via the Kolmogorov-Smirnov (K-S) test. For the statistics of nonparametrical data Chi-Square was deployed.

## 3. Results

A total of 676 consecutive blunt head injury patients with 1 or more CT scans and a neurosurgery consultation were studied. The mean age of all 676 cases was 34.38 ± 23.09 (95% CI 32.63 to 36.12) (range: 0–102). 462 were male. A total 325 patients were sent home without control CT scans. The mean age of these was 37.32 years ± 24.16 (95% CI 34.69 to 39.96). 351 patients received a control CT. Their mean age was 31.65 ± 21.74 (95% CI 29.37 to 33.93). Of these 351 patients, 105 were under 18 years old (pediatric age group) (72 were males and 33 females). The mean age of the pediatric population was 7.29 ± 5.04 (95% CI 6.31 to 8.27). Of the 351 patients, 246 were ≥18 years old (182 males and 64 females). Their mean age was 42.05 ± 17.37 (95% CI 39.08 to 44.23). A fixed protocol was followed, and the period between the first and the repeated CT scan was 6 hours ± 13 minutes. The indications for repeat CT scan were not clear, they were taken according to the neurosurgeon's clinical decision.

The general characteristics of patients who had control cranial CTs are given in Table 1. When these 351 control CTs were analyzed, it was seen that there were no different pathologies in 346 (98.6%) of them. In CTs of the other 3 (0.9%) patients, there were increases in or new pathological condition, in other 2 (0.6%) there was a decrease (Table 2). There were no pathological findings in 293 (83.5%) of the patients whose first CTs were normal. Of 24 (6.8%) patients who had a hemorrhage in first CT, only 1 showed an increase in the hemorrhage; there was a decrease in the pathological signs in 2. Of 27 (7.7%) patients who showed a fracture in first CT, 2 had new intracranial hemorrhage. There was a correlation between presence of underlying disease (medical or neurological) and change in second CTs; it was statistically significant (*P* < 0.001, *χ* ^2^ test), but when we searched the correlation of the type of illness and change in CTs, due to the small number of the groups, this correlation was not statistically significant.

When the pediatric subgroup was analyzed, it was seen that there were no changes between first and second CTs.

In our study, there were no significant underlying diseases in the background of 307 (87.5%) patients. From the records of the other 44 patients, there was a chronic medical disease in 37 (84.1%) of them. When we searched underlying medical disease we have seen that there were 4 (COPD) chronic obstructive lung disease, 2 asthma, 11 hypertension, 5 diabetes mellitus, 2 rheumatologic diseases, 1 hyperthyroidism, 1 congestive heart disease, 2 ischemic heart disease, 6 gastrointestinal system disease, 1 schizophrenia, 1 peripheral vascular disease, 1 tuberculosis patients. In the other 6 of 44 patients (13.6%) patients an underlying neurological pathological condition was reported other than intracranial malignancy like epilepsia, old ischemic or hemorrhagic cerebrovascular accident. Only 1 patient had an underlying medical and neurological disease. Of 307 patients who had no chronic disease in their past, one of them showed a decrease in the pathological signs in the second CT; there were no increases or new lesions in second cranial CT in this group. There was statistically significant difference between underlying disease (medical and neurological together) and control cranial CT results in the other group (*P* < 0.001, *χ* ^2^ test). But there was no significant difference between subgroups (neurological group or medical illness group) and control CT changes.

A total of 319 (90.9%) patients were not using any medicine (Table 1). Of 3 patients who had an increase or a new lesion in the second CT only one was using acetyl salicylic acid (ASA) (Table 2). Another two patients were not using any antiaggregant or anticoagulant drug.

Of the patients who were included in this study, 303 (86.3%) of them were sent home, one of them went home voluntarily. A total of 12 (3.4%) patients were taken to the operation room or intensive care unit, 35 were admitted to the wards.

## 4. Discussion

In current practice of head trauma, a CT scan is the initial study of choice to determine the type, extent and severity of traumatic brain injury as well as to determine the management protocol [1]. In acute settings, the cranial CT scan is repeated to assess the progress of an intracranial lesion by neurosurgeons. It is thought that it may alert the clinician for the need for closer observation and also predict the outcome [6]. In affluent societies, professional and societal expectations may also influence the frequency of performance of the serial CT scans. In developing countries such a frequent performance of CT scans is an additional burden on resources, which are always limited. This large, retrospective study demonstrates that patients with head trauma in EDs do not need control cranial CT exams; if in first cranial CT there is no recorded pathological finding the first cranial CTs is diagnostic, and if it is normal then second head CTs do not alter the way that patient will leave the EDs.

The use of scheduled brain CTs for head trauma in patients in the emergency observation room has not been studied much. Their importance/significance remains unclear in the literature. Patients with evidence of traumatic brain injury are typically admitted to the intensive care units or neurosurgery clinics. For the others who are assigned for further observation in the ED, scheduled brain CTs are a common practice to evaluate the progression of any pathological signs in our university hospital. It adds to costs of hospitalization in EDs, increases resource use, exposes patients to additional radiation; and increase; crowding in the ED.

Interventions based on the repeat CT is an important issue, because it may justify the cost of the study if that would improve the outcome for the patient. In studies done on patients with positive initial CT scan, some patients had a change in clinical management [2, 7]. Our study is consistent with previous studies in patients with a positive initial CT scans. But in this study, we found that patients with a normal initial CT scan identified may be safely discharged from the ED.

Some studies clearly show that anticipated or nonneurosurgical management of head trauma, particularly of intracranial hematoma and mass effect has a significant influence on the decision to order a repeat CT scan [6, 8]. However, if there is no clinical deterioration, the repeat CT scan is unlikely to reveal a lesion needing surgical intervention. Additional studies have demonstrated that no interventions were based on repeat CTs unless the patient had coagulopathy, hypotension, intracranial pressure elevation, or a marked neurologic deterioration and concluded that routine scheduled brain cranial CTs are unnecessary. But other studies have reached the opposite conclusion. In our study we found that there are some risks in patients who had underlying disease, but when we searched the correlation of the type of illness and changes in CTs, due to the small number of the subgroups, they were not statistically significant.

Holmes et al. stated that among children with a normal cranial CT scan after minor head trauma, delayed intracranial sequelae requiring intervention are extremely uncommon [9]. In our study, we also see that none of the children had an increased pathological condition in the 2nd CT scan. So our study demonstrates that in children with head trauma, the first cranial CTs are diagnostic, and this age group is under such low risk that there is no need for hospitalization for serial neurologic examination and serial CTs are typically not necessary.

Our study was a retrospective study, so there were inadequate data about progression of patients' Glasgow Coma Scales and symptoms. Also timing of the repeat CT scan and the reasons for the repeated scan may arouse some questions. The exact time period from the injury to the second CT is not clear in this study. Our institution does not have any standing protocols regarding the reason of repeat scans in trauma patients. But scheduled CT scan after 6 hours after the first one is routine program of our neurosurgery clinic. In some studies, it has been advocated that if the first CT scan is done too early after the injury, it can fail to detect the early head injury. They recommended an interval of routine repeat scans to be 12–24 hours; others recommend 8 hours for the repetition [10, 11].

In conclusion, the first CT scan of head trauma patients in EDs are good guides for the status of patients with mild head trauma patients. Repeated CT scans after 6 hours in EDs observation rooms are not necessary if first CT is normal in most situations. Special attention may be needed in patients with underlying chronic disease, patients who had fracture in first CTs, and patients who are taking antiplatelet agents.

## Figures and Tables

**Table 1:** General characteristics of patients who had control cranial CTs.

	No difference in control CT	A new lesion or increase of pathology in control CTs	Decrease of pathology in control CTs	Total
Sex				
Male	251	2	1	254
Female	95	1	1	97
Underlying disease				
None	306	0	1	307
Medical	33	3	1	37
Cranial	6	0	0	6
Cranial and medical	1	0	0	1
Drug use				
None	318	0	1	319
ASA	5	1	0	6
Anticoagulant	4	0	0	4
Others	19	2	1	22
First CT result				
Normal	293	0	0	293
Hemorrhage	21	1	2	24
Fracture	25	2	0	27
Fracture + hemorrhage	4	0	0	4
Other (incidental pathology not related to trauma)	3	0	0	3
Outcome				
Home	301	0	2	303
Ward	33	2	0	35
Operation room and intensive care unit	11	1	0	12
Home voluntarily	1	0	0	1

**Table 2:** General characteristics of patients who had change of pathology in control cranial CTs.

	Age	Sex	Known underlying disease	Medicine use	First CT result	Control CT result	Outcome
Case 1	66	Male	Medical disease	—	Intracranial hemorrhage	Decrease in pathology	Home
Case 2	68	Male	Medical disease	Drug other than antiaggregant or anticoagulant	Fracture	New intracranial hemorrhage	Neurosurgery ward
Case 3	36	Female	Medical disease	Drug other than antiaggregant or anticoagulant	Fracture	New intracranial hemorrhage	Neurosurgery ward
Case 4	60	Male	Medical disease	ASA	Intracranial hemorrhage	Increase in pathology	Operation room
Case 5	57	Female	—	Drug other than antiaggregant or anticoagulant	Intracranial hemorrhage	Decrease in pathology	Home
